# Complexities of complex II: Sulfide metabolism *in vivo*

**DOI:** 10.1016/j.jbc.2022.101661

**Published:** 2022-01-29

**Authors:** Gary Cecchini

**Affiliations:** 1 Molecular Biology Division, San Francisco VA Health Care System, San Francisco, California, USA; 2 Department of Biochemistry & Biophysics, University of California, San Francisco, California, USA

**Keywords:** complex II, succinate dehydrogenase, sulfide quinone oxidoreductase, fumarate reductase, hydrogen sulfide, electron transport, coenzyme Q, hypoxia, mitochondria, complex I, NADH-quinone oxidoreductase, complex II, succinate-quinone oxidoreductase or succinate dehydrogenase, SDH, complex III, ubiquinol-cytochrome c reductase, complex IV, cytochrome c oxidase, CoQ, coenzyme Q_10_, *i.e.*, ubiquinone, DHODH, dihydroorotate dehydrogenase, FRD, fumarate reductase, SQOR, sulfide quinone oxidoreductase, TCA, tricarboxylic acid cycle

## Abstract

High levels of H_2_S produced by gut microbiota can block oxygen utilization by inhibiting mitochondrial complex IV. Kumar *et al.* have shown how cells respond to this inhibition by using the mitochondrial sulfide oxidation pathway and reverse electron transport. The reverse activity of mitochondrial complex II (succinate-quinone oxidoreductase, *i.e.*, fumarate reduction) generates oxidized coenzyme Q, which is then reduced by the mitochondrial sulfide quinone oxidoreductase to oxidize H_2_S. This newly identified redox circuitry points to the importance of complex II reversal in mitochondria during periods of hypoxia and cellular stress.

It is generally accepted that mitochondria evolved from an endosymbiotic prokaryote (1). In eukaryotes, the chemical reactions of oxidative phosphorylation, the Krebs (tricarboxylic acid, TCA) cycle, and fatty acid oxidation are almost exclusively found within the mitochondrion. In mammals, the mitochondrion is considered to require oxygen as an electron acceptor to function for ATP production. In numerous lower eukaryotes, however, anaerobically functioning mitochondria are found which can use alternate electron acceptors (2). These lower eukaryotes and bacteria use specialized enzymes (*e.g.*, quinone- or NADH-dependent fumarate reductase, nitrate reductase, etc.) and alternative electron carriers (*e.g.*, rhodoquinone, menaquinone, etc.) to generate ATP and maintain metabolism. It could therefore be posited that mammalian mitochondria retain catalytic functions normally associated with lower eukaryotes that reside in anaerobic or microaerophilic environments.

Kumar *et al.* (3) have recently provided an interesting example of how mammalian intestinal epithelial cell mitochondria adapt to high levels of H_2_S produced environmentally by the microbiome by coupling sulfide quinone oxidoreductase (SQOR) and respiratory complex II (succinate dehydrogenase, SDH) activities. Sulfide quinone oxidoreductase and complex II are flavoproteins that are bound to the inner mitochondrial membrane, and both enzymes reduce coenzyme Q (CoQ) in their normal physiological activities. Complex II is unique in that it is the only membrane-bound component of the TCA cycle, but it is also a key component of the electron transport chain, providing reducing equivalents through ubiquinone reduction that are used by complex III (ubiquinol-cytochrome c reductase) and complex IV (cytochrome c oxidase) for oxidative phosphorylation. At high concentrations, H_2_S can act as electron transport chain toxin, inhibiting oxygen utilization by tightly binding to the heme of respiratory complex IV (cytochrome *c* oxidase) (4). Because H_2_S at lower concentrations is involved in cell signaling, it is of interest to determine how H_2_S is cleared from the cell when the mitochondrial respiratory chain is inhibited.

The approach of Kumar *et al.* (3) used a variety of methodologies, including nanodisc-embedded SQOR, ectopic expression of enzymes to manipulate the NAD^+^/NADH ratio and the products of the malate–aspartate shuttle in cell lines, metabolomics, shRNA knockdown of expression of enzymes involved in CoQ reduction, and knockdown of the flavoprotein subunit of complex II (SDHA) in mouse models. The authors show that SQOR can indeed use oxygen as an electron acceptor; however, the reaction is ∼1000-fold less efficient than when CoQ is the electron acceptor. Previously, this same group had shown that H_2_S inhibition of complex IV caused a reductive shift in the NAD^+^/NADH ratio (5), implicating the involvement of mitochondrial complex I (NADH-quinone oxidoreductase), which uses NADH to reduce CoQ. Here, the authors confirmed that cellular H_2_S oxidation was linked to the mitochondrial NAD(P)H redox pool.

The authors also used metabolomic profiling to reveal changes in glycolytic, TCA cycle, and purine metabolism intermediates in HT29 cells after exposure to inhibitory levels of H_2_S. Importantly, succinate, a TCA cycle substrate and inflammatory marker, was significantly increased in four of five cell lines tested, and the levels of another TCA cycle intermediate malate were significantly lower. Because succinate is the substrate of complex II (*i.e.*, succinate + CoQ → fumarate + CoQH_2_), the authors queried whether the buildup of succinate was because of complex II working in reverse (*i.e.*, fumarate + CoQH_2_ → succinate + CoQ). Here, the authors used a clever approach by adding the clinically approved and membrane permeable compound dimethyl fumarate to the cell lines to increase intracellular fumarate levels. This significantly increased H_2_S oxidation, consistent with the idea that complex II was working in reverse. These findings were confirmed by using complex II specific inhibitors and knockdown of the catalytic SDHA subunit of complex II. Intriguingly, the higher fumarate levels in the cell also shortened the recovery time for relief of H_2_S inhibition, but not when SDHA was knocked down. A key finding in the article was made by looking at cellular H_2_S-dependent oxygen consumption rates in knockdowns of complexes I and II at low levels of H_2_S; here it was shown that complex II helps clear sulfide by catalyzing the oxidation of CoQH_2_. In other words, by working in reverse, complex II generates CoQ, thus providing this substrate for SQOR. Studies in SDHA knockout mice, where intestinal epithelial cells environmentally exposed to high levels of H_2_S were targeted, confirmed these results. They also demonstrated that because the malate–aspartate shuttle and purine nucleotide cycle are potential sources of mitochondrial fumarate (6) that manipulating these pathways perturbed H_2_S oxidation, consistent with reversal of complex II.

Although many years ago, it was shown that SDH could catalyze fumarate reduction, this reaction was much less efficient than succinate oxidation (7), making it unclear whether the reaction was sufficiently robust to support the reverse activity of complex II in mitochondria *in vivo*. The reason the reverse reaction is less efficient likely arises from thermodynamic (*i.e.*, the redox potential between CoQH_2_ and FAD) and kinetic barriers. It has been shown that *Escherichia coli* complex II could work in reverse to support anaerobic cell growth (8), although this requires significant overexpression of the enzyme complex. Therefore, it is gratifying that the findings described above regarding sulfide metabolism and complex II are supported by recent findings by Spinelli *et al.* (9) in mammalian mitochondria, showing that fumarate is used as a terminal electron acceptor and that indeed complex II does work in reverse *in vivo*. Both of these articles (3, 9) are consistent with a model where increased levels of reduced CoQH_2_ are generated in the mitochondrion by complex I and other CoQ-reducing enzymes during environmental conditions/stresses (hypoxia, toxins, etc.) where the utilization of CoQH_2_ is inhibited. What is clear is that increased levels of reduced CoQH_2_ and fumarate are the prerequisite for complex II to work in reverse (*i.e.*, fumarate reduction).

As shown by Kumar *et al.*, (3) it is now apparent that the fumarate reductase activity of complex II with concomitant oxidation of CoQH_2_ works in concert with SQOR in a redox circuit used to oxidize H_2_S. Complex II reversal now appears to be present in most mammalian tissues (9) and may even be linked to reperfusion injury after tissue ischemia (6). The ability of complex II to work in reverse may also help sustain pyrimidine nucleotide metabolism under conditions of hypoxia where oxygen may be limiting, such as in tumors. This is consistent with observations that knocking out complex II activity perturbs pyrimidine metabolism in breast cancer cells (10). Further work is needed to establish the specific directionality of complex II under different types of metabolic stress. In summary, these recent reports affirm the importance of complex II in mitochondrial respiration and metabolism and suggest that looking for other functional redox partners such as SQOR could be a useful endeavor.

## Conflict of interest

The author declares that he has no conflicts of interest with the contents of this article.
